# Loofah-Inspired Hierarchical Omniphobic Membrane for Efficient Dissolved Gas Extraction

**DOI:** 10.3390/polym18151798

**Published:** 2026-07-23

**Authors:** Wei Zhang, Haifeng Gao, Xuran Zhu, Yanzong Meng, Leyu Shen, Zhongyao Jiang, Hongjian Gao

**Affiliations:** China Electric Power Research Institute, Beijing 100192, China; zhuxuran@tju.edu.cn (X.Z.); 18369903886@163.com (Y.M.); ly540253825@163.com (L.S.); angelyao_000@163.com (Z.J.); jangseohee1987@aliyun.com (H.G.)

**Keywords:** loofah-inspired, omniphobic membrane, hierarchical structure, spraying-deposition, oil-gas separation, dissolved gas extraction

## Abstract

To address the persistent challenge of membrane wetting during oil-gas separation in transformer condition monitoring, an omniphobic composite membrane was developed to facilitate the reliable online detection of dissolved gases. An F-CNTs/Teflon AF/PVDF composite membrane, featuring a loofah-like hierarchical structure and omniphobic properties, was fabricated via spraying-deposition strategy on the polyvinylidene fluoride (PVDF) substrate. The morphology, surface chemical composition, wettability and stability of the F-CNTs/Teflon AF/PVDF composite membrane were systematically characterized. Subsequently, the oil-gas separation performance of the composite membrane was evaluated using standard transformer oil containing dissolved gases as the feed solution. The results indicated that fluorinated carbon nanotubes (F-CNTs) were successfully modified onto the membrane surface, creating a re-entrant morphology composed of an intersecting nanotube network that mimics the hierarchical architecture of a loofah. The F-CNTs/Teflon AF/PVDF composite membrane exhibited exceptional omniphobicity, achieving contact angles of 168.2 ± 1.5° and 127.5 ± 1.0° towards DI water and mineral insulating oil, respectively. Additionally, the loofah-inspired composite membrane demonstrated robust thermal and ultrasonic stability. In oil-gas separation tests, the omniphobic membrane displayed a rapid response and high efficiency for dissolved gas extraction, achieving dynamic equilibrium within 64 min. Furthermore, the modification improved permeation efficiency by 25.6%. These results suggest that the developed omniphobic membrane is a promising alternative for oil-gas separation in the condition monitoring of oil-filled electrical equipment.

## 1. Introduction

Driven by the ‘dual carbon’ goals, efforts are being accelerated to build a new power system with renewable energy as the mainstay [1,2]. The large-scale grid integration of intermittent renewable energy sources, including wind and solar power, poses unprecedented challenges to grid flexibility and equipment reliability [3,4]. As the core hub equipment in power transmission and distribution systems, transformers are critical to grid security, with their operational health directly linked to system stability [5]. In oil-immersed transformers, insulating oil functions as both an insulating and cooling medium [6,7,8,9]. Over prolonged operation, the insulating oil and solid insulation materials gradually deteriorate, generating characteristic gases such as hydrogen (H_2_), methane (CH_4_), ethane (C_2_H_6_), ethylene (C_2_H_4_), acetylene (C_2_H_2_), carbon monoxide (CO) and carbon dioxide (CO_2_) [10,11,12,13]. In current practice, C_2_H_2_ is universally considered the most representative fault gas, and its concentration variations serve as critical indicators of the type and severity of internal faults in oil-immersed electrical equipment [14,15,16].

Dissolved gas analysis (DGA) is universally acknowledged as a premier and widely adopted technique for fault diagnosis in oil-immersed power equipment [17]. The conventional DGA workflow consists of four sequential stages: oil sampling, oil-gas separation, gas detection, and fault criteria analysis. As a critical step, oil-gas separation extracts characteristic gases dissolved in the insulating oil and then transfers them to the detection unit [18,19]. Its separation efficiency and response speed directly dictate the overall detection accuracy and real-time performance of the DGA system. Currently, international standards such as IEC 60599 and IEEE Std C57.104 recommend DGA for transformer condition assessment, establishing concentration thresholds and ratio criteria for typical fault gases [20,21].

Depending on the separation mechanisms, oil-gas separation technologies are primarily categorized into traditional mechanical degassing methods and membrane separation [22,23,24,25]. As an emerging alternative to conventional mechanical methods (e.g., vacuum, dynamic headspace and oscillation degassing), membrane separation exploits the permeation properties of specialized membranes. Driven by the solution-diffusion mechanism, it facilitates continuous mass transfer from the oil phase to the gas phase, enabling efficient gas separation and purification. This technology possesses remarkable advantages, including a compact structure, simple operation, the elimination of waste oil treatment, low energy consumption, and low maintenance costs [26,27,28]. Additionally, its ease of integration and miniaturization allows for direct coupling with detection units to achieve continuous online monitoring without the risk of volatile oil vapor contaminating the detection chamber [28,29,30]. Consequently, it has attracted widespread attention in online DGA monitoring applications.

The oil-gas membrane separation process is motivated by the partial pressure difference arising from the temperature and concentration gradients across the membrane [29,30,31]. The membrane plays a central role in this process. The choice and structural design of membrane materials directly determine the separation efficiency and the permeate quality. An optimal oil-gas separation membrane should integrate high gas permeation flux, an appropriate equilibration time and superior resistance to oil swelling and fouling [30,31]. To date, a variety of polymeric materials, including polyvinylidene fluoride (PVDF), polytetrafluoroethylene (PTFE), polydimethylsiloxane (PDMS), Teflon AF2400, polyimide (PI), and fluorinated ethylene propylene (FEP), have been widely investigated for the fabrication of oil-gas separation membranes [32,33,34]. Among them, Teflon AF2400, an amorphous perfluorinated polymer, is recognized as an ideal candidate due to its high fractional free volume, favorable gas permeability, and excellent chemical inertness [34]. To mitigate the high cost of Teflon AF2400, researchers have favored the development of composite membranes that utilize Teflon AF2400 as the selective layer, thereby reducing overall membrane fabrication costs. Han et al. [35] developed a composite membrane by depositing Teflon AF2400 onto a ceramic ultrafiltration support, achieving an 8 μm selective layer and an oil-gas equilibration time of 5 h. Lu et al. [36] fabricated an omniphobic PVDF-based composite membrane via silica nanoparticle deposition and Teflon AF2400 coating, demonstrating robust performance in liquid-gas separation. However, existing oil-gas separation membranes still suffer from critical bottlenecks in practical applications. The prolonged equilibration time hinders their ability to promptly respond to the rapid detection demands of sudden transformer faults. Consequently, enhancing permeation flux and reducing equilibration time without compromising high selectivity have become core issues that urgently need to be resolved in online DGA monitoring.

Current studies on oil-gas separation membranes have predominantly aimed at improving efficiency through separation layer thinning or the development of high-permeability materials. However, the critical aspects of mitigating interfacial temperature polarization and enhancing interfacial heat transfer remain largely unexplored. It has been demonstrated that the incorporation of emerging functional materials, such as graphene and nanotube derivatives, into the membrane matrix is a promising strategy for fabricating composite membranes [37,38]. By tailoring the interfacial physicochemical microenvironment, this approach effectively overcomes the limitations of conventional membranes and boosts their permeation performance. Herein, we propose a novel strategy to develop omniphobic membranes embedded with thermally conductive functional materials, thereby intensifying the heat and mass transfer at the oil-gas interface. The primary objective is to simultaneously enhance separation performance by mitigating interfacial temperature polarization and bolster anti-wetting properties through constructing an omniphobic surface.

In this work, an F-CNTs/Teflon AF/PVDF composite membrane with a loofah-like hierarchical structure and omniphobic characteristics was developed via a spraying-deposition strategy. Specifically, a Teflon AF layer was coated onto a PVDF substrate, followed by the immobilization of CNTs@PDA and simultaneous fluorosilane modification. The CNTs@PDA was synthesized by modifying carbon nanotubes with PDA to introduce abundant amine and phenolic hydroxyl active sites, thereby facilitating subsequent hydrophobic modification. The membrane morphological features, surface chemical composition, wetting behavior and stability under harsh conditions were characterized systematically. Moreover, oil-gas separation performance and the equilibrium time were assessed using standard transformer oil containing C2H2 as the feed solution.

## 2. Experimental Setup

### 2.1. Materials and Chemicals

The multi-walled carbon nanotubes (MWCNTs, outer diameter: 30–50 nm, length: 0.5–2 μm, purity: >95%) were supplied by Nanjing XFNANO Materials Technology Co., Ltd., Nanjing, China. Fluorinert^®^ FC-40 was provided by 3M (China) Co., Ltd., Shanghai, China. Amorphous fluoropolymer Teflon AF2400X-J was purchased from Chemours Chemical (China) Co., Ltd., Shanghai, China. Poly(dimethylsiloxane) (PDMS, hydroxy terminated), dopamine hydrochloride (≥98%), hydrochloric acid (HCl, 37%), sodium hydroxide (NaOH), anhydrous ethanol (99.7%), isopropanol (IPA, 99.5%), tris (hydroxymethyl) aminomethane/hydrochloric acid buffer solution (1 M, pH = 8.5) and 1H,1H,2H,2H-perfluorotetradecyltriethoxysilane (CF_3_(CF_2_)_11_(CH_2_)_2_Si(OC_2_H_5_)_3_, PFTES, ≥97%) were gained from Shanghai Aladdin Biochemical Technology Co., Ltd., Shanghai, China. Cyclohexane (≥99%) was sourced from Shanghai Macklin Biochemical Technology Co., Ltd., Shanghai, China. Mineral insulating oil was obtained from Petro China Co., Ltd., Beijing, China. A flat sheet PVDF membrane (Durapore^®^, 0.1 μm) was supplied by Merck Millipore Co., Ltd., Darmstadt, Germany. Deionized water (DI) was produced from an ultrapure water purification system (Millipore) with resistivity exceeding 18.0 MΩ·cm. The certified standard gas mixture was gained from Air Products (China) Co., Ltd., Shanghai, China. All chemicals and reagents were utilized directly without further purification.

### 2.2. Fabrication of F-CNTs/Teflon AF/PVDF Composite Membrane

(1) Synthesis of polydopamine-modified carbon nanotubes (CNTs@PDA)

Briefly, 0.5 g MWCNTs was dispersed in 100 mL of anhydrous ethanol via magnetic stirring for 60 min and ultrasonication for 30 min at room temperature. Afterwards, 200 mL tris (hydroxymethyl) aminomethane-hydrochloric acid buffer solution (Tris-HCl, 50 mM, pH = 8.5) was added to the dispersion, with the assist of NaOH solution. The mixture was then magnetically stirred for 2 h, followed by ultrasonication for 30 min. Subsequently, 600 mg dopamine hydrochloride was introduced into the above mixture and stirred vigorously at ambient temperature for 12 h to ensure complete reaction. The blended solution was separated by centrifugation and washed with deionized water and ethanol repetitiously until approached neutrality. The resulting precipitate was collected and vacuum-dried overnight at 60 °C to obtain black powder, named as CNTs@PDA nanocomposite.

(2) F-CNTs/Teflon AF/PVDF composite membrane with sandwich structure

After being dried at 100 °C for 12 h, amorphous fluoropolymer Teflon AF2400X-J (Teflon AF) was dissolved in fluorinert^®^ FC-40 with continuous stirring, yielding the homogeneous solution at a concentration of 1.0 wt%. Thereafter, 2 mL of the Teflon AF solution was sprayed onto the PVDF substrate using a distance of 20 cm and a pressure of 0.35 MPa. The modified PVDF membrane was transferred to a vacuum oven and dried at 25 °C for 3 h to achieve the Teflon AF/PVDF composite membrane.

**Figure 1:**
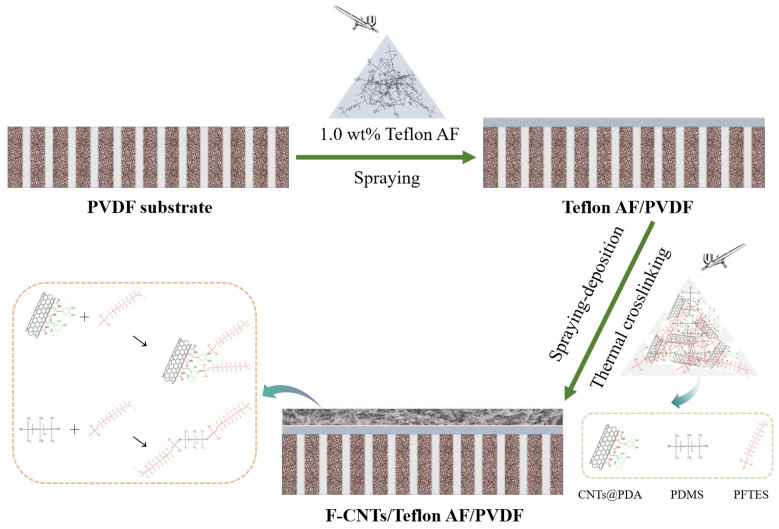
The dehydration reactions among diverse molecules and the fabrication process of the F-CNTs/Teflon AF/PVDF composite membrane.

Then, 0.05 g CNTs@PDA was ultrasonically dispersed in 10 mL isopropanol at room temperature for 90 min to obtain a stable mixture, designated as liquid A. Separately, PDMS was dissolved in 5 mL cyclohexane via magnetic stirring for 3 h, followed by ultrasonication for 60 min at 25 °C to acquire liquid B. Afterwards, liquid B was combined with liquid A, and 0.5 mL PFTES was added dropwise. The mixture was continuously stirred and subjected to ultrasonication at 50 °C for 6 h to achieve a homogeneous suspension. The resulting dispersion was spray-deposited onto a Teflon AF/PVDF composite substrate under controlled pressure and working distance to construct a well-defined sandwich architecture. Subsequently, the coated membrane was transferred to a vacuum oven and thermally treated at 125 °C for 4 h to facilitate the cross-linking and curing of the coating layer. The modified Teflon AF/PVDF membrane was referred as F-CNTs/Teflon AF/PVDF composite membrane, based on the content of PDMS (0.1%, 0.2%, 0.4%, 0.6% and 0.8%). The straightforward preparation procedure for the F-CNTs/Teflon AF/PVDF composite membrane is depicted in Figure 1.

### 2.3. Membrane Characterization

The morphologies of the CNTs and CNTs@PDA samples were characterized by a transmission electron microscopy (TEM, JEOL JEM-2100F, Tokyo, Japan) equipped with an energy dispersive spectrometer (EDS, Kevex, San Carlos, CA, USA) at an accelerating voltage of 200 kV. The surface and cross-section morphologies of membrane samples were performed via scanning electron microscopy (SEM, Hitachi S-4800, Tokyo, Japan), preceded by an ion sputtering instrument (Hitachi E-1045, Tokyo, Japan). Membrane surface chemical composition was determined by attenuated total reflectance Fourier transform infrared spectroscopy (ATR-FTIR, Bruker TENSOR II, Berlin, Germany) with a 4 cm^−1^ resolution across the scanning range of 4000–400 cm^−1^ wavenumbers. Membrane surface chemical structure was further analyzed by X-ray photoelectron spectroscopy (XPS, Thermo Scientific Nexsa G2, Waltham, MA, USA) with an Al Kα radiation source (hʋ = 1486.6 eV), covered with a scanning range of 0–1200 eV and a 400 μm spot size. High-resolution spectra for C1s, O1s and F1s were obtained in a 50.0 eV pass energy analytical mode. Membrane surface wettability was quantitatively assessed using an optical contact angle goniometer (DataPhysics OCA 15EC, Filderstadt, Germany) equipped with a video camera in the static sessile drop mode [39]. All data were collected under controlled ambient conditions.

### 2.4. Membrane Stability and Oil–Gas Separation Experiments

The membrane stability was assessed via overheating and ultrasonic treatment. In detail, the membrane was immersed in the mineral insulating oil at 90 °C for 3 d, followed by sonication at 50 °C for 6 h. A comparative study of the surface morphology and wettability was conducted before and after harsh condition treatment.

**Figure 2:**
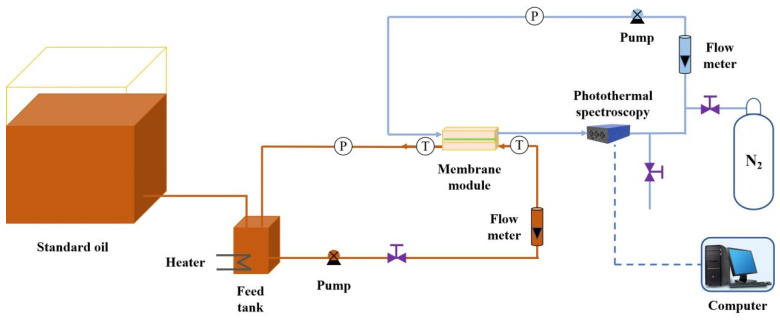
Schematic diagram of the oil-gas separation experimental setup.

The oil-gas separation experiment was performed using a cross flow membrane module under atmospheric pressure. A schematic diagram of the oil-gas separation test setup is shown in Figure 2. The effective membrane area was 11.5 cm^2^. Before testing, membrane samples were stabilized for 2 h with the pure mineral insulating oil under steady circulation. During the testing, the oil flow rate was set at 30 L/h. The feed pressure and temperature were maintained at 0.15 ± 0.01 MPa and 50 ± 0.5 °C, respectively. The feed was sourced from the dissolved gas online monitoring system calibrating apparatus (Zhongfen JD-LA, Shangqiu, China), in accordance with the requirements of the Q/GDW 10536-2021 Technical Specification for Dissolved Gas Online Monitoring Device in Transformer Oil [40]. The permeate was kept at ambient temperature and pressure. The effective volume of the gas chamber was approximately 1.0 mL. The target product gas was collected and enriched on the permeate with the operating time. The gas concentration was detected by the lab-scale spectroscopy system equipped with an optical-thermal effect detector in real time.

The membrane separation performance was evaluated as a function of operating time under conditions of constant gas concentration, where the corresponding time was the maximum. Once the separation time reached 90% of the maximum value, the oil-gas separation was considered to be in an equilibration state. A shorter equilibrium time indicated superior membrane separation performance. The detailed derivation of the formula is provided in the Supplementary Materials. The equilibrium time is described by Equation (1) as follows [41]:

(1) $$t_{eq}=\frac{2.3Vd}{10^{5}HA}$$

where *t*_eq_ is the equilibrium time (s), *V* is the effective volume of the gas chamber (m^3^), *d* is the thickness of the membrane (m), *H* is the permeate coefficient (m^2^·s^−1^·Pa^−1^), and *A* is the effective area of the membrane (m^2^).

## 3. Results and Discussion

### 3.1. Characterizations of CNTs@PDA Powder

**Figure 3:**
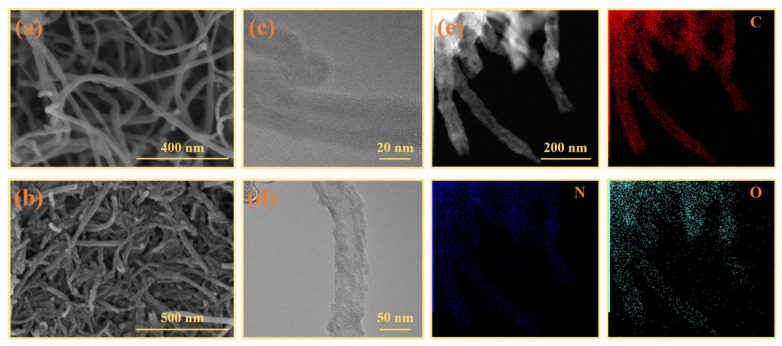
SEM micrographs of (**a**) CNTs and (**b**) CNTs@PDA, TEM micrographs of (**c**) CNTs and (**d**) CNTs@PDA, (**e**) EDS mapping images of C, N and O element distributions on the CNTs@PDA.

The morphological structures of CNTs and CNTs@PDA are investigated via SEM, TEM and EDS element mapping to confirm the PDA modification on the carbon nanotubes. The SEM and TEM surface morphologies of CNTs and CNTs@PDA along with C, N and O element distributions of EDS mapping in the CNTs@PDA are exhibited in Figure 3. As observed, significant changes occurred on the surface of pristine CNTs and the surface became rougher due to the coating, resulting in an increased diameter. In a Tris-HCl buffer solution at pH = 8.50, dopamine hydrochloride undergoes self-polymerization under an oxygen atmosphere to generate polydopamine (PDA). This process effectively encapsulated the CNTs within the PDA coating, thereby introducing abundant amine and phenolic hydroxyl active sites. Furthermore, N and O elements were clearly revealed on the CNTs@PDA by EDS mapping images, providing strong evidence for the successful PDA modification.

### 3.2. Characterizations of F-CNT/Teflon AF/PVDF Composite Membrane

The grafting of PFTES onto the CNTs@PDA surface was achieved via hydrolysis, driven by fluorosilanization and hydrogen bonding. The hydroxyl-terminated PDMS not only served as a binder to anchor the CNTs@PDA but also underwent grafting modification with the hydrolyzed PFTES. Consequently, the surface energy of both CNTs@PDA and PDMS was significantly reduced due to the PFTES modification. After spray coating, the carbon nanotube layer was firmly attached to the membrane surface, leading to the successful fabrication of F-CNTs/Teflon AF/PVDF composite membranes. According to the content of PDMS (0.1%, 0.2%, 0.4%, 0.6% and 0.8%), F-CNTs/Teflon AF/PVDF composite membranes were recorded as F-CNTs/Teflon AF/PVDF-1, F-CNTs/Teflon AF/PVDF-2, F-CNTs/Teflon AF/PVDF-3, F-CNTs/Teflon AF/PVDF-4 and F-CNTs/Teflon AF/PVDF-5, respectively. Subsequently, the properties of composite membranes were evaluated through comprehensive characterizations of their morphology, elemental composition, chemical structure, and surface wettability.

**Figure 4:**
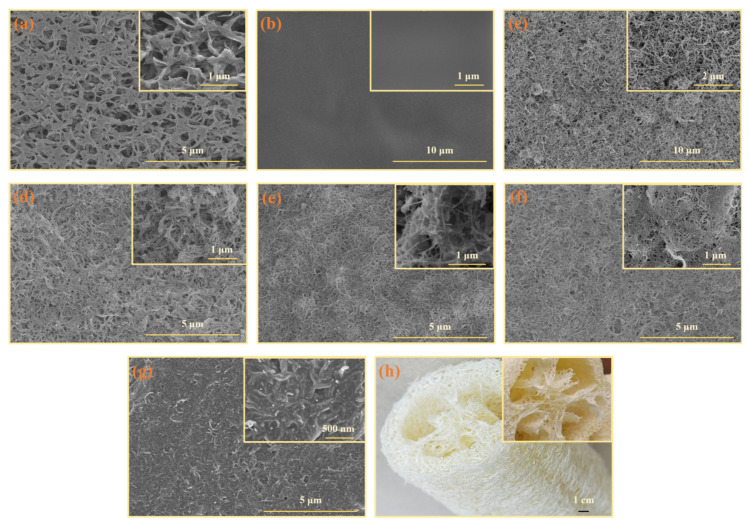
SEM surface micrographs of pristine and composite membranes: (**a**) pristine PVDF membrane, (**b**) Teflon AF/PVDF membrane, (**c**) F-CNTs/Teflon AF/PVDF-1 membrane, (**d**) F-CNTs/Teflon AF/PVDF-2 membrane, (**e**) F-CNTs/Teflon AF/PVDF-3 membrane, (**f**) F-CNTs/Teflon AF/PVDF-4 membrane and (**g**) F-CNTs/Teflon AF/PVDF-5 membrane, with the corresponding images at a higher magnification being shown in the upper right corner; (**h**) digital photos of loofah sponge.

The SEM surface morphologies of the pristine PVDF membrane, the Teflon AF/PVDF composite membrane and the F-CNTs/Teflon AF/PVDF composite membranes with varying PDMS contents are presented in Figure 4a–g. A comparison revealed significant differences in the surface morphology between composite membranes and the pristine PVDF membrane. Following the deposition of Teflon AF, a uniform and dense functional layer with a smooth feature was formed on the membrane surface, as shown in Figure 4b. The CNTs-modified composite membranes indicated distinct surface morphologies. When the content of PDMS was low, intertwined CNTs were observed on the membrane surface. As the PDMS content increased in the spraying dispersion, the gaps among the CNTs were filled with PDMS. An interwoven loofah-like sponge structure was constructed, and numerous island-like protrusions were built on the membrane surface, as depicted in Figure 4e. They are beneficial for enhancing the omniphobic performance. However, with a further increase in the PDMS concentration, the CNTs were adhesively carpeted and even completely submerged. This not only enhanced mass transfer resistance during the membrane separation process but also hindered the construction of desirable re-entrant structures.

**Figure 5:**
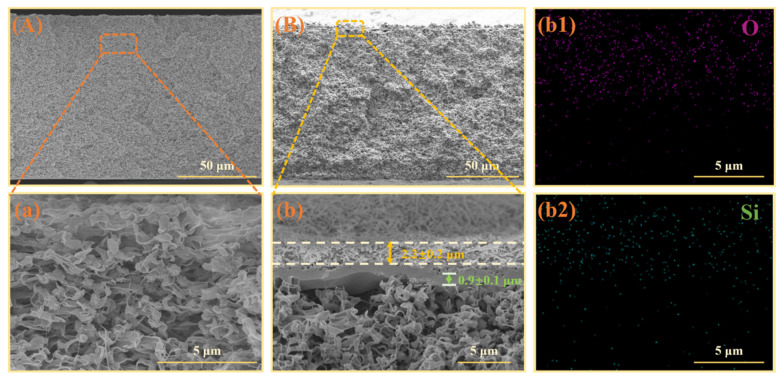
SEM cross-sectional micrographs: (**A**,**a**) pristine PVDF membrane, (**B**,**b**) F-CNTs/Teflon AF/PVDF-3 membrane and (**b1**,**b2**) the corresponding EDS mapping images of O and Si element distributions on the cross-section of F-CNTs/Teflon AF/PVDF-3 membrane.

The SEM cross-sectional morphologies of the pristine PVDF membrane and the F-CNTs/Teflon AF/PVDF-3 membrane are displayed in Figure 5A,a,B,b. The corresponding EDS mappings of O and Si elements on the cross-section of the F-CNTs/Teflon AF/PVDF-3 membrane are presented in Figure 5(b1,b2). As observed, compared to that of the pristine membrane, the internal pore structure of the F-CNTs/Teflon AF/PVDF-3 membrane exhibited no significant change, retaining its characteristic sponge-like pores. The modification with Teflon AF and fluorinated CNTs was confined strictly to the membrane surface. As indicated in Figure 5b, the thicknesses of the Teflon AF layer and the fluorinated CNTs coating were measured at 0.9 ± 0.1 μm and 2.2 ± 0.2 μm, respectively. It is evident that the O element is distributed throughout both the Teflon AF and fluorinated CNTs coating, whereas the Si element is exclusively localized within the fluorinated CNTs coating, originating from the fluorosilane and PDMS. These results confirm that the CNTs and Teflon AF were modified on the PVDF membrane without affecting the internal pore structure.

Surface morphology and chemical composition play pivotal roles in enhancing membrane performance. As demonstrated by the aforementioned morphological characterizations, hierarchical micro/nano papillae structures were successfully constructed on the composite membrane surface. The resultant rough surface significantly contributes to the improvement of membrane performance. To further elucidate the chemical composition of the membranes, ATR-FTIR and XPS characterizations were subsequently conducted.

**Figure 6:**
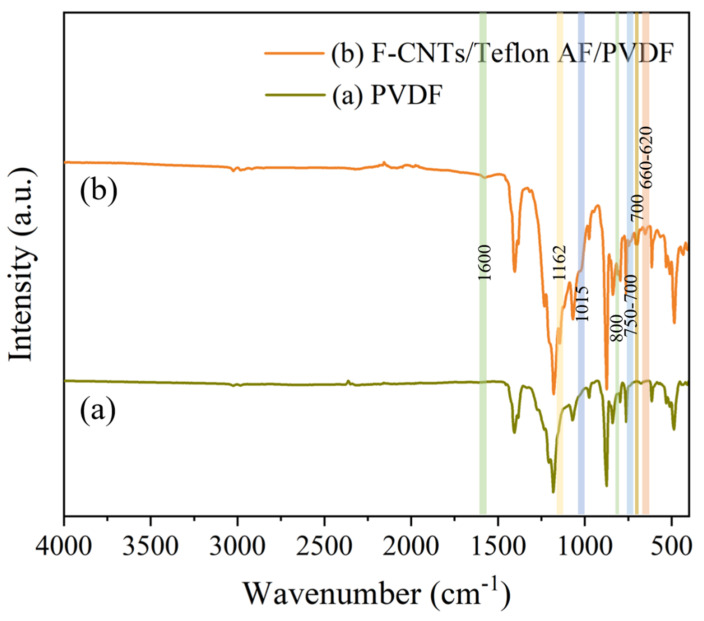
ATR-FTIR spectra of pristine PVDF membrane and F-CNTs/Teflon AF/PVDF membrane.

The ATR-FTIR spectra of the pristine PVDF membrane and the F-CNTs/Teflon AF/PVDF membrane are shown in Figure 6. Compared to the pristine PVDF membrane, the F-CNTs/Teflon AF/PVDF membrane exhibited several new characteristic absorption peaks. Specifically, the band at 1620–1600 cm^−1^ was attributed to the C=C stretching vibration of benzene rings, arising from the CNTs@PDA layer on the membrane surface [42]. New peaks at approximately 1162 cm^−1^ and 700–750 cm^−1^ were ascribed to the bending vibrations of C-F covalent bonds in -CF_3_ groups [43]. Additionally, the peak observed in the 1100–1000 cm^−1^ region was assigned to the stretching vibration of Si-O-C covalent bonds [42], confirming that the hydrolyzed PFTES underwent a dehydration condensation reaction with the PDA-modified CNTs. Two new absorption peaks at 800 cm^−1^ and 700 cm^−1^ were ascribed to the stretching vibrations of Si-C covalent bonds, while the peak at 660–620 cm^−1^ was corresponded to Si-O-Si stretching vibrations [42]. The appearance of these new peaks confirms that the CNTs@PDA was successfully modified by fluorosilane and deposited onto the surface of the F-CNTs/Teflon AF/PVDF composite membrane.

**Figure 7:**
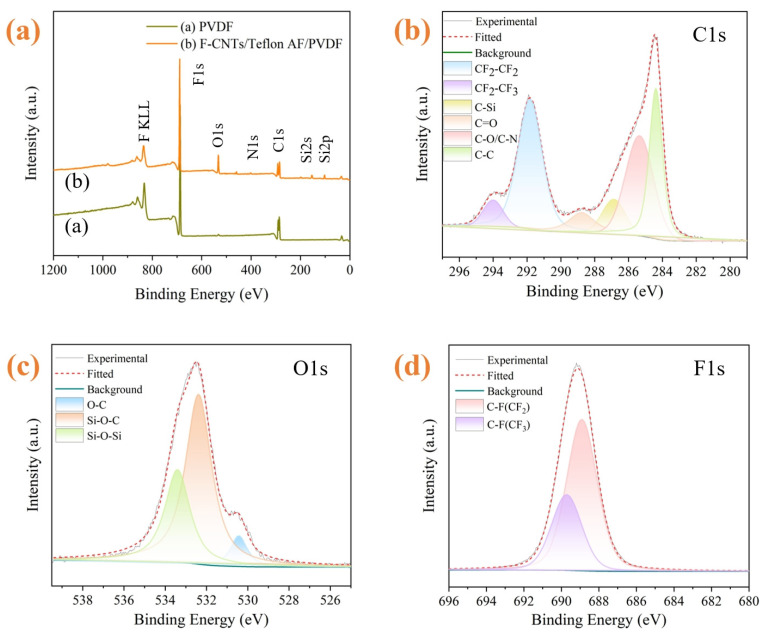
XPS spectra of pristine PVDF membrane and F-CNTs/Teflon AF/PVDF membrane: (**a**) 3D full spectra of membranes, (**b**) C1s fitting narrow spectrum of the F-CNTs/Teflon AF/PVDF membrane, (**c**) O1s fitting narrow spectrum of the F-CNTs/Teflon AF/PVDF membrane, (**d**) F1s fitting narrow spectrum of the F-CNTs/Teflon AF/PVDF membrane.

X-ray photoelectron spectroscopy (XPS) was employed to characterize the chemical composition and analyze the chemical bonding states of the membrane surfaces. The XPS survey spectra and high-resolution narrow-scan spectra for pristine PVDF membrane and F-CNTs/Teflon AF/PVDF membrane are presented in Figure 7. As shown in Figure 7a, the survey spectrum of the F-CNTs/Teflon AF/PVDF membrane reveals the emergence of new characteristic peaks assigned to O 1s, N 1s, Si 2s and Si 2p. Following the surface modification, the elemental composition of the membrane surface experienced significant changes, as detailed in Figure S1. In particular, the atomic content of N increased from 0 to 1.83%, Si rose from 0 to 5.66%, and O reached 11.3%. These variations provide strong evidence for the successful surface deposition of CNTs@PDA and the subsequent grafting modification with the fluorosilane PFTES.

Representative peaks at binding energies of 293.8 eV, 291.8 eV, 288.9 eV, 286.3 eV, 285.4 eV and 284.5 eV were detected in the C 1s high-resolution spectrum of the F-CNT/Teflon AF/PVDF membrane (Figure 7b), which are assigned to CF_2_-CF_3_, CF_2_-CF_2_, C=O, C-Si, C-O/C-N and C-C bonds, respectively [44,45]. The O 1s spectrum was deconvoluted into three characteristic peaks at 533.5 eV, 532.5 eV and 530.4 eV, attributed to Si-O, C-O-Si and C-O bonds, respectively [44,45]. Additionally, the F 1s spectrum was fitted with two characteristic peaks corresponding to C-F bonds, originating from CF_2_-CF_2_ and CF_2_-CF_3_ groups, as displayed in Figure 7d. These high-resolution spectra confirm the chemical states of the major elements on the composite membrane surface. Specifically, the characteristic peaks ascribed to C=O and C-N bonds verify the presence of CNTs@PDA. Meanwhile, the signals derived from CF_2_-CF_3_, C-O-Si, Si-O and C-Si bonds demonstrate the successful surface modification with PFTES. Mechanistically, the hydrolyzed fluorosilane initially interacts with the hydroxyl groups on the CNTs@PDA surface and the terminal hydroxyl groups of PDMS via hydrogen bonding. Subsequently, a dehydration condensation reaction occurs under high-temperature thermal treatment, leading to the stable immobilization of the low-surface-energy fluorosilane on the composite membrane surface.

### 3.3. Properties of F-CNTs/Teflon AF/PVDF Composite Membrane

**Figure 8:**
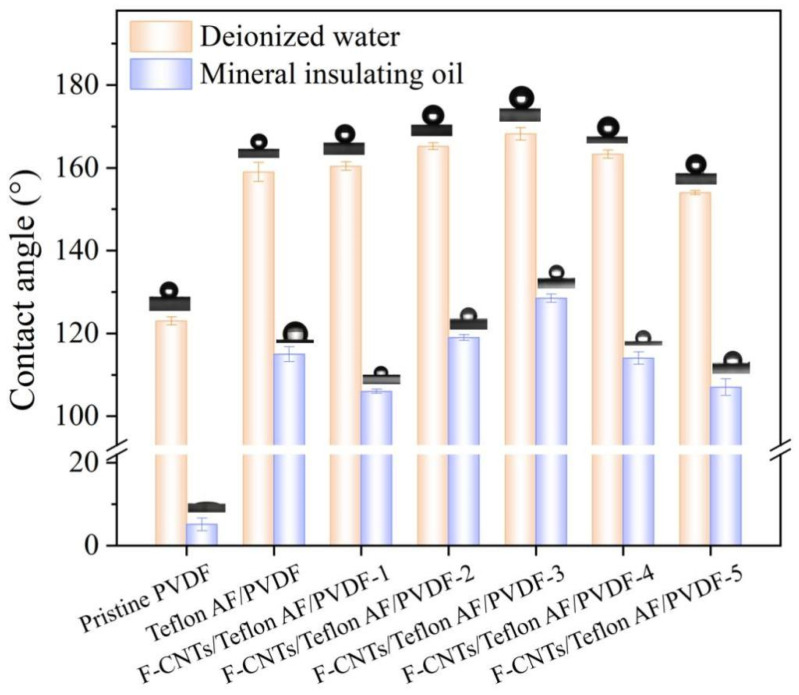
Contact angles and the corresponding droplet photographs of pristine PVDF membrane, Teflon AF/PVDF membrane and F-CNTs/Teflon AF/PVDF membranes; standard deviations were obtained from at least three different measurements.

Membrane wettability is critical to its oil-gas separation performance. To evaluate the surface wettability of the pristine PVDF and composite membranes, contact angle measurements were conducted using deionized water and mineral insulating oil. The resulting contact angles and the corresponding droplet profiles on the membrane surfaces are illustrated in Figure 8. Owing to the inherent hydrophobic nature of PVDF, the pristine PVDF membrane exhibited a high water contact angle of approximately 123.0°, whereas the contact angle for insulating oil was only 5.1 ± 1.5°. Once in contact with the PVDF membrane, the oil droplets spontaneously spread and penetrated into the membrane pores, resulting in a wicking phenomenon that rendered the membrane surface nearly superoleophilic. This behavior stems from the weak omniphobicity of PVDF, making the pristine membrane vulnerable to oil wetting and fouling during oil-gas separation operations.

After modification with Teflon AF2400, the Teflon AF/PVDF membrane exhibited significantly higher water and oil contact angles. This enhancement is attributed to the low surface energy (15.6 mN/m) of the Teflon AF2400 coating. After the functionalization with F-CNTs and PFTES, the F-CNTs/Teflon AF/PVDF membrane surface was introduced with intertwined CNTs featuring a loofah-like structure and low-surface-tension perfluorosilane (16.2–17.0 mN/m). Notably, this surface tension is much lower than that of mineral insulating oil in air at room temperature (~27 mN/m). The F-CNTs/Teflon AF/PVDF-3 membrane exhibited a water contact angle of ~168.2° and an insulating oil contact angle of ~127.5°, confirming its superhydrophobic and oleophobic characteristics. Such excellent omniphobicity endows the membrane with robust resistance to wetting and fouling during oil-gas separation. Nevertheless, slight variations in contact angles were observed across the F-CNTs/Teflon AF/PVDF membranes due to micro-morphology differences, yet its hydrophobic and oleophobic properties remained superior to those of the pristine membrane.

**Figure 9:**
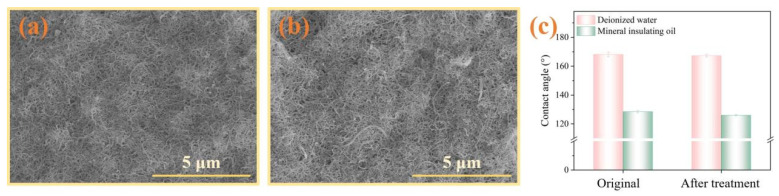
SEM microscopic images and surface contact angles of F-CNTs/Teflon AF/PVDF-3 composite membrane before and after heating and ultrasonication: (**a**) original photographs, (**b**) heating and ultrasonic treatment, (**c**) the contact angle variation before and after treatment.

Membrane stability is a critical factor in long-term oil-gas separation operations. The changes in surface micro-morphology and contact angles of the F-CNTs/Teflon AF/PVDF-3 composite membrane after thermal and ultrasonic treatments are presented in Figure 9. Even after immersion in hot oil at 90 °C and subsequent ultrasonication, the composite membrane retained its hierarchical micro-nano rough morphology characterized by island-like protrusions. The contact angles of deionized water and mineral insulating oil on the membrane surface were approximately 167.5° and 126.0°, respectively. These values remained highly consistent with the pre-immersion test results without significant degradation, demonstrating the robust stability of the omniphobic composite membrane under harsh conditions.

### 3.4. F-CNTs/Teflon AF/PVDF Composite Membrane Separation Performance

Drawing upon the analysis of surface morphology and wetting behavior, we propose the anti-wetting mechanisms for both the Teflon AF/PVDF membrane and the omniphobic F-CNTs/Teflon AF/PVDF membrane. The preceding results revealed that identical droplets exhibited distinct configurations on the pristine PVDF, Teflon AF/PVDF, and F-CNTs/Teflon AF/PVDF-3 membranes. This clearly indicated that the wetting properties of the membranes were dictated by the surface features, particularly the geometric topography and the surface free energy.

On the pristine PVDF membrane, the wetting behavior of water droplets resided in a transitional state between the Wenzel and Cassie–Baxter states. The actual solid-liquid contact area exceeded the apparent value, indicating a relatively weak resistance to wetting. Conversely, water droplets on the Teflon AF/PVDF membrane were in the Cassie–Baxter state, exhibiting superior anti-wetting properties. For the F-CNTs/Teflon AF/PVDF-3 membrane, the interlaced nanotubes constructed a re-entrant surface morphology, while the island-like protrusions formed by the nanotubes further enhanced the surface roughness. The creation of this hierarchical structure not only increased surface roughness but also enlarged the voids to create a vapor phase environment, allowing more air to be trapped within the re-entrant structure. When a water droplet fell on the composite membrane, the air layer at the solid-liquid-gas triple-phase interface hindered wetting, thereby reducing the solid-liquid contact area and increasing the liquid-gas contact area. Meanwhile, the underlying air cushion elevated the energy barrier and resistance against the droplet intrusion into membrane pores. Consequently, droplets remained in a thermodynamically metastable Cassie–Baxter state on the F-CNTs/Teflon AF/PVDF-3 membrane, enabling them to roll off freely with high mobility. Ultimately, the F-CNTs/Teflon AF/PVDF-3 composite membrane demonstrated exceptional anti-wetting capability and robust omniphobic performance.

**Figure 10:**
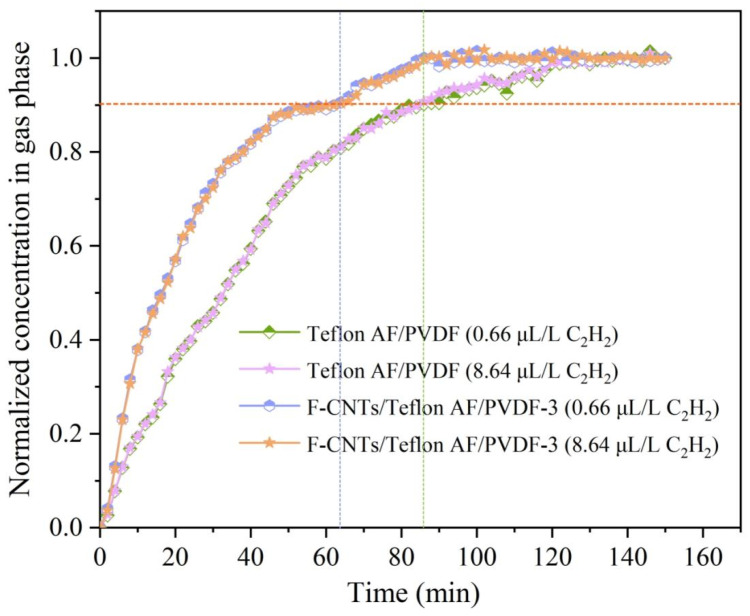
The gas permeable test in oil/gas separation process for Teflon AF/PVDF and F-CNT/Teflon AF/PVDF-3 composite membranes. The horizontal dashed line represents the equilibrium state, and the vertical dashed lines represent the equilibrium time.

Insulating oil dissolved with acetylene (C_2_H_2_), a representative dissolved gas, was used as the feed. Different concentrations of feed were selected with 0.66 μL/L (low concentration) and 8.64 μL/L (medium concentration). During the testing process, the feed was maintained at a constant flow rate. The variation in gas concentration on the permeate side for both the Teflon AF/PVDF membrane and the F-CNTs/Teflon AF/PVDF-3 membrane is illustrated in Figure 10. It is evident that as the operation time progressed, the gas concentration on the permeate side of membranes gradually increased and eventually stabilized within a certain range. Notably, the equilibrium time for oil-gas separation remained essentially consistent with the same membrane across different oil concentrations. This finding provides theoretical evidence that the equilibrium time is independent of the feed concentration. The Teflon AF/PVDF membrane required 86 min to reach equilibrium, whereas the F-CNTs/Teflon AF/PVDF-3 membrane achieved dynamic equilibrium across both sides in just 64 min. This modification with the CNTs coating increased by 25.6% for the permeation efficiency of the composite membrane. Instead, the pristine PVDF membrane underwent rapid wetting, indicating its failure in oil-gas separation performance.

During the oil-gas separation process, a temperature polarization layer developed at the interface between the insulating oil and the membrane. This phenomenon compromised the driving force for the degassing of dissolved gases, thereby prolonging the time required to reach the equilibrium state. It is well established that the thermal conductivity of carbon nanotubes (CNTs) is approximately 3000 W/m·K, vastly exceeding that of the polymeric Teflon AF2400 (0.18–0.22 W/m·K) [46,47,48,49]. The superior thermal conductivity of the CNTs coating on the F-CNTs/Teflon AF/PVDF-3 membrane facilitates rapid heat transfer from the bulk hot oil to the oil-gas separation interface. This effectively mitigates temperature polarization at the membrane surface and enhances the transmembrane vapor pressure difference induced by the temperature gradient. Consequently, the driving force for oil-gas separation is intensified, which shortens the equilibrium time and improves overall efficiency. Furthermore, the interlaced nanotubes construct a loofah-like intertwined architecture containing abundant voids. This structural feature significantly reduces gas diffusion resistance, enabling the rapid release of dissolved gases at the interface and forming a high-concentration gas accumulation zone within the biomimetic loofah voids. Subsequently, the gases permeate to the downstream side via the solution-diffusion mechanism through the Teflon AF2400 selective layer, thereby achieving efficient oil-gas separation. Additionally, Table 1 presents a comparison of oil-gas separation performance between the membrane developed in this study and those reported in the literature. For instance, a ceramic membrane tube modified with a 1.8 wt% Teflon AF2400 coating (8 μm thickness) required 5 h to reach separation equilibrium. Therefore, compared to the Teflon AF/PVDF membrane and the literature, the F-CNTs/Teflon AF/PVDF-3 membrane exhibits a significantly shorter equilibrium time (64 min) and superior oil-gas separation efficiency, demonstrating its promising potential for condition monitoring of oil-filled electrical equipment.

## 4. Conclusions

The F-CNTs/Teflon AF/PVDF composite membrane with a loofah-like hierarchical structure and omniphobicity was prepared by a versatile spraying-deposition method. Specifically, the Teflon AF layer was coated onto a PVDF substrate, followed by the immobilization of heat-conducting CNTs@PDA and simultaneous fluorosilane modification. Comprehensive characterization confirmed that a re-entrant morphology with a hierarchical rough architecture was successfully constructed on the membrane surface, characterized by island-like protrusions and an interwoven loofah-like sponge structure. Concurrently, fluoroalkyl silane was grafted onto the membrane to impart ultra-low surface energy. The synergistic effect of this re-entrant architecture and ultra-low surface energy endowed the F-CNTs/Teflon AF/PVDF-3 composite membrane with robust omniphobic characteristics, exhibiting super-repellency towards deionized water (168.2°) and mineral insulating oil (127.5°). Furthermore, the optimal composite membrane demonstrated excellent thermal and ultrasonic stability under harsh conditions. In oil-gas separation tests, the F-CNTs/Teflon AF/PVDF-3 membrane achieved dynamic equilibrium for extracting dissolved C_2_H_2_ from insulating oil in just 64 min, a significantly shorter time compared to the pristine Teflon AF/PVDF membrane. Integrating omniphobicity with interfacial heat conduction, this membrane facilitates efficient dissolved gas extraction from insulating oil, holding great potential for online monitoring of oil-filled equipment.

## Figures and Tables

**Table 1:** Comparison of membrane performance in oil/gas separation application.

Membrane Substance	Modification Method	Treated Membrane	Omniphobicity	Feed Composition	Temperature Difference (°C)	Oil-Gas Separation Equilibrium Time (h)	Ref.
Ceramic membrane tube (7.5 mm inner diameter, 12.5 mm outer diameter, 100 mm length and 50 nm pore size)	coating by 1.8 wt% Teflon AF2400	Teflon AF2400	---	transformer oil contained H_2_, CO_2_, CO, CH_4_, C_2_H_4_, C_2_H_6_ and C_2_H_2_	20	5	[35]
PAI hollow fiber membrane	vacuum-assisted dip-coating by 1.0 wt% Teflon AF2400 solution	Teflon/PAI	156.3 ± 1.8° and 123.0 ± 2.5° towards DI water and mineral insulating oil, respectively	insulating oil solution contained 16.84 μL·L^−1^ C_2_H_4_, 3.64 μL·L^−1^ C_2_H_2_, 24.01 μL·L^−1^ C_2_H_6_, 12.46 μL·L^−1^ CH_4_, 1475.29 μL·L^−1^ CO_2_, 457.24 μL·L^−1^ CO and 65.94 μL·L^−1^ H_2_	50 ± 1	2	[41]
Flat sheet membrane	spraying-deposition by heat-conducting CNTs and Teflon AF2400 solution	F-CNTs/Teflon AF/PVDF	~168.2° and ~127.5° towards DI water and mineral insulating oil, respectively	insulating oil solution contained 0.66 μL·L^−1^ C_2_H_2_ or 8.64 μL·L^−1^ C_2_H_2_	50 ± 1	~1	This work

## Data Availability

Data are contained within the article.

## Supplementary Materials

The following supporting information can be downloaded at https://www.mdpi.com/article/10.3390/polym18151798/s1: Figure S1: Surface element compositions of pristine PVDF membrane and F-CNTs/Teflon AF/PVDF membrane.
