# Supplementary material for: Loofah-Inspired Hierarchical Omniphobic Membrane for Efficient Dissolved Gas Extraction
Source: Polymers (Basel). 2026 Jul 23;18(15):1798. doi: 10.3390/polym18151798 (PMC13468637; doi:10.3390/polym18151798)
Supplement: Supplementary file 1 [file polymers-18-01798-s001.zip › polymers-4423705-supplementary.pdf]

Loofah-inspired hierarchical omniphobic membranes for efficient dissolved gas extraction

Wei Zhang *, Haifeng Gao *, Xuran Zhu, Yanzong Meng, Leyu Shen, Zhongyao Jiang and Hongjian Gao

China Electric Power Research Institute, Beijing 100192, China

* *Correspondence:18739968007@163.com (W.Z.); gaommv@163.com (H.G.); Tel.: +86-22-2740-7854 (W.Z.); Fax: +86-57-8795-1225 (W.Z.)*

Element (%)	PVDF	F-CNTs/Teflon AF/PVDF
C1s	50.15	37.39
F1s	49.85	43.83
O1s	---	11.3
Si	---	5.66
N1s	---	1.83

Figure. S1 Surface element compositions of pristine PVDF membrane and F-CNTs/Teflon AF/PVDF membrane

The bidirectional diffusion of gas in the membrane was conducted until reaching a dynamic equilibrium, with the gas concentration in the permeate remaining constant. Gas permeation of oil and gas phases on both sides of the separation membrane was complied with the following relationship [1]:

$$C_g = \ (9.87KC_0 - C_{g0}) \ \times \left[1 - \exp\left(\frac{-10^5 HAt}{dV}\right)\right] + C_{g0} \qquad (1\text{-}1)$$

where C_{g0} and C_g are the initial concentration and the concentration when the permeation time is t in the gas chamber at 100 kPa, respectively (μL·L^{-1}), C_0 is the dissolved gas concentration in the oil at 100 kPa (μL·L^{-1}), K is the equilibrium constant in the oil-gas separation process, H is the permeate coefficient (m^2·s^{-1}·Pa^{-1}), A is the effective area of membrane (m^2), d is the thickness of membrane (m), V is the volume of gas chamber (m^3), and t is the permeation time (s).

When the oil-gas separation process reached the equilibrium state, the membrane and the gas chamber structure had no influence on the gas volume fraction. However, the membrane and the gas chamber structure affected the equilibrium time. The structural parameter was denoted as b, which was calculated by the following equation [1, 2]:

$$b = \frac{10^5 HA}{dV} \qquad (1\text{-}2)$$

During the initial stage, there was only background gas in the gas chamber, and the permeate gas concentration was 0, namely C_{g0}=0. The equation (1-2) was simplified as:

$$C_g = 9.87KC_0[1 - \exp(-bt)] \tag{1-3}$$

C_{max} was the maximum value of gas concentration in the gas chamber, namely C_{max}=$9.87KC_0$. The gas concentration was indicated as:

$$\mathrm{C_g} = \mathrm{C_{max}}[1 - \exp(-\mathrm{bt})] \tag{1-4}$$

In practice, when time was long enough until reaching the equilibrium state in the oil-gas separation process, the equilibrium time was recorded with t_{90}. It was explained as the time for 90% of the maximum value C_{max}. t_{90} was obtained from equation (1-2) and equation (1-4) as follows:

$$t_{90} = \frac{2.3Vd}{10^5 HA} \tag{1-5}$$

As shown in equation (1-5), the oil-gas separation equilibrium time t_{90} was closely related to *A*, *d* and *V*. It was proportional to the gas chamber volume and the membrane thickness. With the increase of membrane effective contact area, the oil/gas separation equilibrium time decreased.

References

[1] Han, Y.; Ding, F.; Hao, C.; Lv, S.; Wang, X. The oil-gas separation characteristics of ceramic/Teflon AF2400 composite membrane. Separation and Purification Technology, 2012, 88, 19-23.

[2] Song, H.; Wang, H.; Wang, H.; Liu, Y.; Liu, S.; Chen, C. Rapid evaluation method for oil-gas separation membrane utilizing mass spectrometry. Chinese Journal of Analytical Chemistry, 2025, 53.5, 100515-100523.
